# Association between cyclin-dependent kinase 4/6 inhibitors and nephrotoxicity in patients with breast cancer: A Systematic Review and meta-analysis

**DOI:** 10.1016/j.isci.2024.111370

**Published:** 2024-11-12

**Authors:** Jiayong Cui, Jinquan Sun, Xueying Zhou, Yi Li, Jiuda Zhao, Guoshuang Shen

**Affiliations:** 1 Breast Disease Diagnosis and Treatment Center, Affiliated Hospital of Qinghai University, Xining, China

**Keywords:** Health sciences, Medicine, Medical specialty, Internal medicine, Oncology

## Abstract

Nephrotoxic adverse events (AEs) have been observed in patients with breast cancer receiving cyclin-dependent kinase (CDK) 4/6 inhibitors. This study aimed to evaluate the risk of nephrotoxicity associated with these inhibitors through a meta-analysis of 17 randomized controlled trials involving 19,638 patients. The results indicate a significant increase in all-grade nephrotoxic AEs, including elevated blood creatinine levels, acute kidney injury, and renal impairment (RR = 3.12, 95% CI [2.11, 4.63]). The incidence of grade 3 or higher nephrotoxicity was also more prevalent among treated patients (RR = 3.12, 95% CI [1.74, 5.58]). Subgroup analyses revealed varying risks among 4 different CDK 4/6 inhibitors. Furthermore, analysis of FDA Adverse Event Reporting System data corroborated these findings, emphasizing the occurrence of nephrotoxicity in real-world settings. Clinicians should remain vigilant in monitoring renal function indicators when prescribing CDK4/6 inhibitors.

## Highlights

• Analysis of 17 RCTs shows higher nephrotoxic AEs with CDK4/6 inhibitors
• FAERS data indicates potential kidney function compromise in real-world use
• Increased vigilance is needed in monitoring renal function during treatment
• Use these drugs with caution in older patients or those with kidney-related issues

## Graphical abstract

**Figure:**
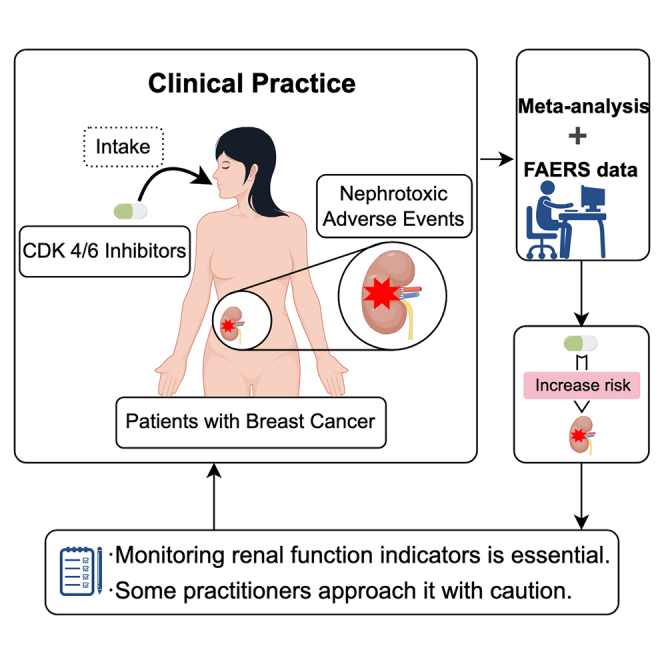

## Introduction

Breast cancer has surpassed lung cancer to become the most common and highest incidence malignant tumor among women globally.^1^ Hormone receptor-positive (HR+) and human epidermal growth factor receptor 2-negative (HER2-) breast cancer represent a common subtype,^2^ and endocrine therapy (ET) is one of the primary treatment modalities.^3^ However, a significant proportion of patients with HR+/HER2-breast cancer develop resistance to ET, and to address this clinical challenge, researchers have begun to identify new therapeutic targets and drugs, leading to the emergence of cyclin-dependent kinase (CDK) 4/6 inhibitors.^4^^,^^5^

There are currently four oral CDK 4/6 inhibitors approved for use in patients with HR+/HER2-breast cancer: palbociclib, ribociclib, abemaciclib, and dalpiciclib.^6^^,^^7^^,^^8^^,^^9^^,^^10^ Multiple studies have validated the effectiveness of the combination of above CDK 4/6 inhibitors with ET in HR+/HER2-breast cancer, demonstrating prolonged progression-free survival and overall survival, alongside reduced recurrence risk compared to ET alone.^11^^,^^12^^,^^13^^,^^14^^,^^15^^,^^16^ Globally, the combination of CDK 4/6 inhibitors with ET has become the standard of care for HR+/HER2-breast cancer.^17^^,^^18^ Despite the proven efficacy of CDK 4/6 inhibitors, nephrotoxic events have also occurred in some patients with breast cancer treated with CDK 4/6 inhibitors as the clinical utilization of these drugs continues to rise.^19^^,^^20^ In addition, a study confirmed that acute kidney injury in six biopsies-confirmed patients with HR+/HER2-breast cancer was directly related to CDK4/6 inhibitors.^21^ All of these highlight the importance of timely identification and management of renal toxicity during CDK 4/6 inhibitor therapy to mitigate potential renal dysfunction or impairment. However, the risk of nephrotoxic adverse events (AEs) associated with CDK4/6 inhibitors remains undetermined at present.

## Results

### Eligible studies and characteristics

Our initial search yielded 3,368 reports. After removing obvious duplicates and screening titles and abstracts, we reviewed 225 studies for full-text screening. Finally, 17 articles from electronic databases were included after excluding duplicate RCT studies, involving a total of 19,638 patients (Figure S1).

Table 1 illustrates the composition of this meta-analysis, encompassing 15 phase III studies^22^^,^^23^^,^^24^^,^^25^^,^^26^^,^^27^^,^^28^^,^^29^^,^^30^^,^^31^^,^^32^^,^^33^^,^^34^^,^^35^^,^^36^ and 2 phase II studies.^37^^,^^38^ Among the 17 trials included, palbociclib was employed in 8 trials, abemaciclib in 4 trials, ribociclib in 3 trials, and dalpiciclib in 2 trials. Fourteen studies examined patients with advanced breast cancer comprising one study focusing on patients with metastatic breast cancer, while 3 studies investigated patients with early breast cancer. Furthermore, 15 studies enrolled patients with Eastern Cooperative Oncology Group (ECOG) performance status scores of 0–1, whereas 2 studies included patients with ECOG performance status scores of 0–2. All studies were RCTs, and adequate randomization procedures were reported in all studies, except for one study with potential issues regarding randomized stratification. Among the included trials, 3 open-label studies lacked blinding of either treatment administration or outcome assessment, while the remainder was double-blinded. The risk of bias for each study is delineated in Supplementary Material (Figure S2).

**Table 1:** Characteristics of eligible studies

Author (Year)	NCT number	Acronym	Phase	Cancer staging	ECOG	Patient groups		Age		Nephrotoxicity-related events					
								Mean/Median (SD/Full Range)		Patients for safety analysis		All-grade		≥3 grade or SAEs	
						T (N)	C, (N)	T	C	T (N)	C, (N)	T (N)	C, (N)	T (N)	C, (N)
Masakazu Toi,^33^ (2023)	NCT02107703	MONARCH 2	III	advanced breast cancer	0–1	Abemaciclib 150mg + Fulvestrant (446)	Placebo +Fulvestrant (223)	59.3(11.2)	61.1(11.7)	441	223	71	1	8	0
Masato Takahashi,^32^ (2022)	NCT02246621	MONARCH 3	III	advanced breast cancer	0–1	Abemaciclib 150mg + NSAI (328)	Placebo +NSAI (165)	63.13(9.92)	62.92(9.96)	327	161	77	7	10	0
Ana Ganfornina Andrades et al.,^24^ (2024)	NCT03155997	MONARCH E	III	early breast cancer	0–1	Abemaciclib 150 mg + ET (2808)	ET alone (2829)	52.20(11.26)	52.10(11.20)	2791	2800	285	18	8	1
Qing Yuan Zhang,^31^ (2020)	NCT02763566	MONARCH plus	III	advanced breast cancer	0–1	Abemaciclib 150mg + NSAI (207)	Placebo +NSAI (99)	56.4(10.8)	55.5(9.8)	205	99	25	3	1	1
						Abemaciclib 150mg + Fulvestrant (104)	Placebo + Fulvestrant(53)	59.7(8.5)	58.2(10.3)	104	53	23	1	1	0
Binghe Xu,^36^ (2021))	NCT03927456	DAWNA-1	III	advanced breast cancer	0–1	Dalpiciclib 150mg + Fulvestrant (241)	Placebo + Fulvestrant (120)	50.7(45.3–59.3)	52.4(45.5–60.6)	240	120	25	4	0	0
Pin Zhang,^35^ (2023)	NCT03966898	DAWNA-2	III	advanced breast cancer	0–1	Dalpiciclib 150mg + Letrozole/Anastrozole (303)	Placebo + Letrozole/Anastrozole (153)	54 (47–63)	57 (46–63)	302	153	39	6	0	0
Richard S. Finn,^37^ (2020)	NCT00721409	PALOMA-1	II	advanced breast cancer	0–1	Palbociclib 125mg + Letrozole (84)	Letrozole alone (81)	63 (41–89)	64 (38–84)	83	77	6	5	2	0
Dennis J Slamon,^25^ (2024)	NCT01740427	PALOMA-2	III	advanced breast cancer	0–2	Palbociclib 125mg + Letrozole (444)	Placebo + Letrozole (222)	61.7(10.6)	60.6(11.2)	444	222	38	9	5	1
Sibylle Loibl,^24^ (2021)	NCT01864746	PENELOPE-B	III	early breast cancer	0–1	Palbociclib 125 mg (631)	Placebo (619)	49(22–76)	48(19–79)	633	611	81	67	3	0
Hope S Rugo,^22^ (2021)	NCT01942135	PALOMA-3	III	advanced breast cancer	0–1	Palbociclib 125mg + Fulvestrant (347)	Placebo + Fulvestrant (174)	56.9(11.7)	56.8(10.4)	345	172	21	3	1	0
Miguel Martin (2022)	NCT02028507	PEARL	III	metastatic breast cancer	0–1	Palbociclib 125mg + Exemestane (153)	Capecitabine (143)	60(31–89)	60(38–87)	150	289	36	56	2	3
						Palbociclib 125mg + Fulvestrant (149)	Capecitabine (156)	62(38–86)	60(33–85)	149		39		2	
Binghe Xu,^36^ (2022)	NCT02297438	PALOMA-4	III	advanced breast cancer	0–1	Palbociclib 125mg + Letrozole (169)	Placebo + Letrozole (171)	53.8(8.5)	53.7(9.1)	168	171	10	2	0	0
Georg Pfeiler et al.,^27^ (2023)	NCT02513394	PALLAS	III	early breast cancer	0–1	Palbociclib 125 mg + ET (2884)	ET alone (2877)	52(25–90)	52(22–85)	2841	2902	118	69	4	2
J Albanell (2021)	NCT02690480	FLIPPER	II	advanced breast cancer	0–2	Palbociclib 125mg + Fulvestrant (94)	Placebo + Fulvestrant (95)	64 (38–81)	64 (42–82)	94	94	23	10	0	0
Gabriel N Hortobagyi et a,^30^ (2022)	NCT01958021	MONALEESA-2	III	advanced breast cancer	0–1	Ribociclib 600mg + Letrozole (334)	Placebo + Letrozole (334)	61.4(10.98)	61.9(10.52)	334	330	26	5	3	2
Yen-Shen Lu,^28^ (2022)	NCT02278120	MONALEESA-7	III	advanced breast cancer	0–1	Ribociclib 600mg + NSAI/Tamoxifen + Goserelin (335)	Placebo + NSAI/Tamoxifen + Goserelin (337)	42.6(6.6)	43.7(6.17)	335	337	18	7	1	2
P Neven et al.,^29^ (2023)	NCT02422615	MONALEESA-3	III	advanced breast cancer	0–1	Ribociclib 600mg + Fulvestrant (484)	Placebo + Fulvestrant (242)	63.4(9.78)	62.8(10.59)	483	241	58	11	10	0

NCT: National Clinical Trial; ECOG: Eastern Cooperative Oncology Group; SAEs: severe adverse events; T: Treatment; C: Control; NSAI: nonsteroidal aromatase inhibitor; ET: endocrine therapy.

### Risk of nephrotoxic AEs associated with CDK 4/6 inhibitors

**Figure 1:**
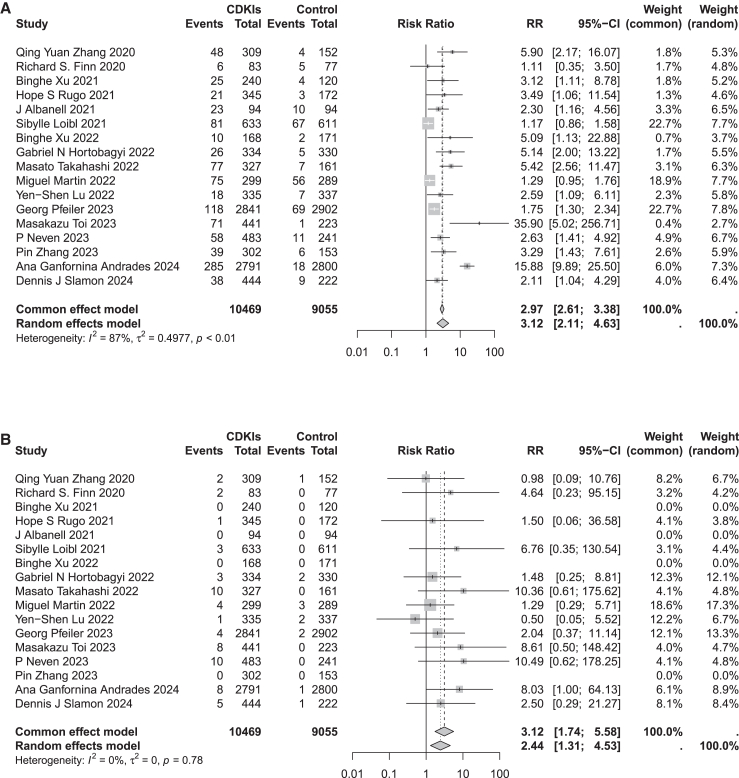
Effects of CDK 4/6 inhibitors on the incidence of nephrotoxic adverse events in patients with breast cancer (A) RR for all-grade nephrotoxicity-related adverse events in the treatment group compared to the control group. (B) RR for grade 3 or higher nephrotoxicity-related adverse events in the treatment group compared to the control group. CDKIs: cyclin-dependent kinases 4/6 inhibitors; RR: risk ratio.

Based on 17 RCTs, the incidence of all-grade nephrotoxic adverse reactions among patients receiving CDK 4/6 inhibitor treatment was 9.73% (1,019/10,469), while it was 3.14% (284/9,055) in the control group. Regarding the risk of grade 3 or higher nephrotoxic adverse reactions, the incidence rate in the treatment group was 0.58% (61/10,469), compared to 0.13% (12/9,055) in the control group. The heterogeneity of AEs across all-grade nephrotoxicity was high, thus a random effects model was utilized. Conversely, there was no significant heterogeneity observed in grade 3 or higher nephrotoxic AEs, leading to the adoption of a common effect model. Compared to the control group, CDK 4/6 inhibitors significantly increased the risk of all-grade nephrotoxic damage (RR = 3.12, 95% CI [2.11, 4.63]), with significant heterogeneity among studies (*I*^2^ = 87%, *p* < 0.01) (Figure 1A). CDK 4/6 inhibitors significantly increased the risk of grade 3 or higher nephrotoxic damage compared to the control group (RR = 3.12, 95% CI [1.74, 5.58]), with no significant heterogeneity among studies (*I*^2^ = 0%, *p* = 0.78) (Figure 1B).

### Subgroup analysis

**Figure 2:**
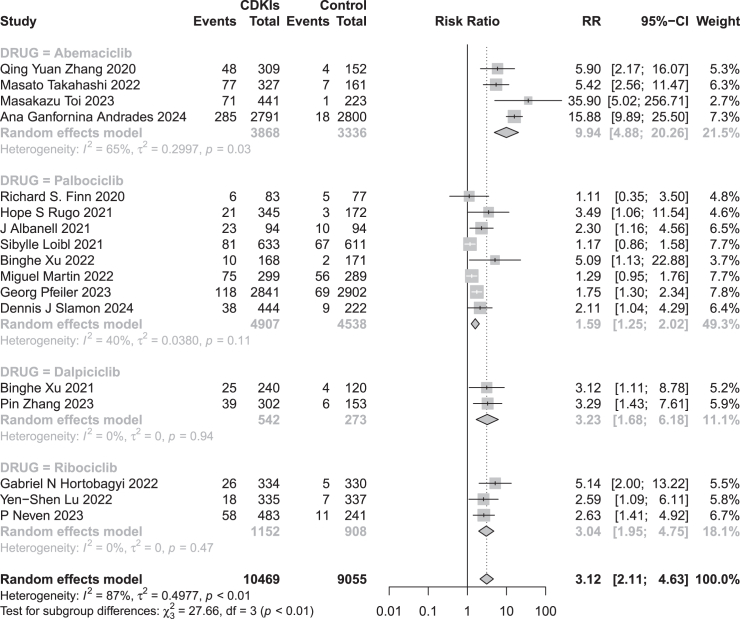
Subgroup analyses of RR according to different CDK 4/6 inhibitors CDKIs: cyclin-dependent kinases 4/6 inhibitors; RR: risk ratio.

We conducted subgroup analyses for nephrotoxic AEs associated with four different CDK 4/6 inhibitors. Stratified by type of CDK 4/6 inhibitor, the incidence rates of all-grade nephrotoxic AEs among patients with HR+/HER2-breast cancer treated with abemaciclib, palbociclib, ribociclib, and dalpiciclib were 10.60% (410/3,868), 7.58% (372/4,907), 8.85% (102/1,152), and 11.81% (64/542), respectively. Compared to the control group, abemaciclib, palbociclib, ribociclib, and dalpiciclib all significantly increased the risk of all-grade CDK 4/6 inhibitor-related nephrotoxic AEs (RR 9.94, 95% CI [4.88, 20.26]; RR 1.59, 95% CI [1.25, 2.02]; RR 3.23, 95% CI [1.68, 6.18]; RR 3.04, 95% CI [1.95, 4.75]), with statistically significant subgroup effects (*p* < 0.01) (Figure 2).

### Sensitivity analysis and publication bias

We conducted a sensitivity analysis of the included studies to assess the stability of the meta-analysis. However, the stepwise exclusion of individual RCTs did not yield significant alterations in the results (Figure S3). Subsequently, a funnel plot was generated to ascertain the presence of any biases in the study results, revealing asymmetry (Figure S4). Furthermore, Egger’s test was used to detect publication bias, revealing *p* < 0.05, suggesting potential publication bias in this study (Figures S5 and S6). Following this, attempts were made to correct publication bias using the trim and fill method, and algorithmic simulations of select unpublished studies were conducted. (Figure S7). Additionally, the Galbraith plot and Baujat plot were constructed to evaluate the impact of each RCT on overall heterogeneity (Figures S8 and S9).

### Basic characteristics of ADE reports in the FAERS database

Adverse drug event (ADE) data related to abemaciclib, palbociclib, ribociclib, and dalpiciclib were extracted from the FAERS database up to the third quarter of 2023. After removing duplicate reports, reports of nephrotoxicity-related ADEs associated with abemaciclib, palbociclib, and ribociclib were obtained. None relevant data on dalpiciclib were retrieved as of April 2024. Basic information on gender, age, and country for nephrotoxicity-related ADEs associated with the three CDK 4/6 inhibitors are presented in Table 2. A total of 162 reports of nephrotoxicity-related ADEs were associated with abemaciclib as the primary suspect drug, with a total of 8,192 ADE occurrences. The median age of reported patients was 68.0, with a mean age of 67.2. Palbociclib was associated with 162 reports of nephrotoxicity-related ADEs, with a total of 7,393 occurrences. The median age of reported patients was 52.5, with a mean age of 69.9. Ribociclib was associated with 182 reports of nephrotoxicity-related ADEs, with a total of 9,770 occurrences. The median age of reported patients was 69.0, with a mean age of 67.3.

**Table 2:** Population characterization of CDK4/6 inhibitor-associated renal toxicity reports in the FAERS database

	abemaciclib	palbociclib	ribociclib
Total	162	162	182
Sex			
Female	159(98.15)	157(96.91)	178(97.80)
Male	3(1.85)	3(1. 85)	3(1.65)
Unknown	0	2(1.24)	1(0.55)
Age			
<50Y	7(4.32)	4(2.47)	15(8.24)
≥50Y&≤75Y	123(75.93)	108(66.67)	122(75.31)
>75Y	32(19.75)	50(30.86)	45(24.73)

**Area & Country**			

Europe			
Germany	6(3.70)	4(2.47)	17(9.34)
France	14(8.64)	14(8.64)	9(4.95)
United Kingdom	2(1.23)	3(1. 85)	19(10.44)
Italy	7(4.32)	1(0.62)	7(3.85)
Other	15(9.26)	0	16(8.79)
North America			
Canada	2(1.23)	8(4.94)	7(3.85)
United States	84(51.85)	86(53.09)	14(7.69)
Other	0	0	6(3.30)
South America			
Argentina	0	13(8.03)	4(2.20)
Brazil	1(0.62)	0	7(3.85)
Colombia	0	0	8(4.40)
Other	0	0	1(0.55)
Asia			
United Arab Emirates	3(1.85)	0	1(0.55)
China	9(5.56)	0	0
Israel	1(0.62)	2(1.24)	11(6.04)
Japan	13(8.02)	12(7.41)	0
India	0	6(3.70)	7(3.85)
Other	3(1.85)	4(2.47)	7(3.85)
Africa			
Egypt	1(0.62)	1(0.62)	4(2.20)
Morocco	0	0	2(1.10)
South Africa	1(0.62)	0	7(3.85)
Oceania			
Australia	0	1(0.62)	0
Unknown	0	0	15(8.24)

### Top 10 nephrotoxicity signals in CDK 4/6 inhibitors

Analysis of nephrotoxicity-related AE signals associated with CDK 4/6 inhibitors yielded the top 10 reported rates and strong signals for “abemaciclib”, “palbociclib”, and “ribociclib” with effective signals ranked in descending order based on preferred terms (PTs) report counts (Table 3).

**Table 3:** The number of abemaciclib, palbociclib and ribociclib nephrotoxicity-related ADE reports in the top 10 PT

	Nephrotoxicity-related PT	N	ROR	PRR
abemaciclib	renal impairment	27	1.014 (0.675, 1.522)	1.014
	renal failure	27	0.744 (0.499, 1.109)	0.745
	blood creatinine increased	22	0.562 (0.363, 0.870)	0.564
	renal disorder	19	1.834 (1.101, 3.054)	1.831
	acute kidney injury	15	0.437 (0.259, 0.738)	0.438
	creatinine renal clearance decreased	5	2.692 (0.948, 7.644)	2.691
	blood creatinine abnormal	5	2.019 (0.739, 5.513)	2.018
	glomerular filtration rate decreased	4	0.861 (0.303, 2.445)	0.861
	chronic kidney disease	2	0.497 (0.118, 2.093)	0.497
	end stage renal disease	1	6.459 (0.404, 103.281)	6.458
palbociclib	blood creatinine increased	21	4.016 (2.562, 6.296)	3.953
	acute kidney injury	14	3.032 (1.760, 5.222)	3.003
	renal impairment	11	2.807 (1.523, 5.172)	2.787
	renal failure	10	1.907 (1.010, 3.5980)	1.897
	glomerular filtration rate decreased	4	6.372 (2.240, 18.12)	6.350
	renal disorder	3	1.724 (0.544, 5.463)	1.721
	creatinine renal clearance decreased	2	6.361 (1.453, 27.853)	6.350
	renal tubular necrosis	2	5.021 (1.168, 21.587)	5.013
	end stage renal disease	1	47.673 (2.98, 762.755)	47.627
	renal injury	1	5.958 (0.744, 47.685)	5.953
ribociclib	blood creatinine increased	41	4.198 (3.003, 5.869)	4.132
	acute kidney injury	27	3.062 (2.046, 4.583)	3.034
	renal impairment	25	3.404 (2.233, 5.190)	3.374
	renal failure	20	1.959 (1.238, 3.097)	1.949
	renal disorder	14	4.592 (2.586, 8.154)	4.566
	glomerular filtration rate decreased	9	8.488 (3.957, 18.209)	8.454
	chronic kidney disease	7	7.852 (3.334, 18.493)	7.828
	blood creatinine abnormal	5	7.355 (2.692, 20.097)	7.339
	renal function test abnormal	4	15.686 (4.423, 55.630)	15.656
	renal injury	1	2.937 (0.367, 23.491)	2.936

PT: preferred term; ROR: reporting odds ratio; PRR: proportional reporting ratio.

## Discussion

This study is the first to explore the nephrotoxicity of CDK 4/6 inhibitors in patients with breast cancer using a meta-analysis and FAERS database. We conducted a comprehensive assessment of the risk of AEs associated with all-grade and grade 3 or higher nephrotoxicity in patients with HR+/HER2-breast cancer treated with CDK 4/6 inhibitors. The findings suggest that patients with HR+/HER2-breast cancer treated with CDK 4/6 inhibitors were at increased risk of nephrotoxic AEs, both in clinical trials and in the real world.

CDK 4/6 inhibitors have become a key drug in the clinical treatment of breast cancer and are often used in conjunction with ET, especially in patients with HR+/HER2-advanced or metastatic breast cancer. These drugs have shown remarkable efficacy in both first-line and follow-up treatment, significantly improving patient survival and quality of life, and firmly establishing their status as cornerstone therapies for breast cancer treatment, and the overall safety profile of CDK4/6 inhibitors is validated by extensive clinical studies. Despite their marked benefits in prolonging progression-free survival, the safety of CDK4/6 inhibitors warrants ongoing scrutiny, particularly in the context of prolonged use. The primary AEs associated with CDK4/6 inhibitors include hematologic toxicities, such as neutropenia, and non-hematologic toxicities, including fatigue, nausea, and diarrhea.^39^ But in clinical practice, patients with breast cancer undergoing treatment with CDK 4/6 inhibitors have sometimes exhibited kidney function impairment indicators, such as blood creatinine increased, during routine monitoring. However, this phenomenon has not raised much clinical concern.

Our findings demonstrate that patients with HR+/HER2-breast cancer treated with CDK4/6 inhibitors experienced a significantly higher incidence of both all-grade and grade 3 or higher nephrotoxic AEs compared to the control group. It is underscored the potential for CDK4/6 inhibitors to lead to clinical outcomes related to nephrotoxicity in patients. Subsequently, we conducted a subgroup analysis of four different CDK4/6 inhibitors. The results revealed a statistically significant association between these inhibitors and nephrotoxic AEs. The RRs of nephrotoxic AEs for the four CDK4/6 inhibitors were 9.94 for abemaciclib, 1.59 for palbociclib, 3.23 for ribociclib, and 3.04 for dalpiciclib. Notably, abemaciclib exhibited the highest RR, consistent with clinical observations of nephrotoxic AEs in patients with breast cancer using this agent.

Notably, diarrhea is more prevalent among patients treated with abemaciclib, and it is demonstrated that advanced age significantly elevates the risk of high-grade diarrhea in these individuals.^40^ Consequently, the administration of abemaciclib in elderly patients necessitates particular caution, as it may lead to dehydration, which can result in diminished intravascular volume and impaired renal perfusion, thereby heightening the risk of kidney damage. Furthermore, electrolyte imbalances may exacerbate pre-existing renal dysfunction. Therefore, in elderly patients experiencing diarrhea, careful monitoring and timely intervention are essential to mitigate the risk of kidney damage.

The precise mechanism underlying nephrotoxic AEs associated with CDK 4/6 inhibitors remains elusive. Nonetheless, various potential pathways have been hypothesized to elucidate how abemaciclib may induce nephrotoxicity. Firstly, the increase in blood creatinine after abemaciclib administration may be due to inhibition of renal transporters. Active secretion of creatinine and other organic cations is at least partially mediated by organic cation transporter 2 (OCT2) in renal tubule basolateral membranes and multidrug and toxin extrusion (MATE) 1 and MATE2-K in proximal tubular apical membranes. Abemaciclib inhibits OCT2 and MATE1/2-K, which causes an increase in blood creatinine.^41^ Secondly, abemaciclib inhibits the transition from the G1 phase to the S phase, resulting in cell-cycle arrest, senescence, and apoptosis. This anticancer activity of abemaciclib may induce apoptosis of kidney-related cells and the release of metabolic by-products, thereby exerting detrimental effects on renal function.^42^ Currently, the mechanism of nephrotoxicity induced by ribociclib is thought to be akin to that of abemaciclib.^43^ *In vitro* experiments, palbociclib was found to inhibit the proliferation and induce senescence of renal tubular epithelial cells, and the senescent secretion phenotype produced by senescent cells may affect the efficacy of tumor therapy and the prognosis of renal function.^44^ However, there is insufficient research or literature on renal damage caused by dalpiciclib.

Significant heterogeneity was observed in AE analyses across all-grade of nephrotoxicity. Sensitivity analysis results indicated that the overall findings remained relatively consistent even upon exclusion of individual studies, suggesting a degree of robustness and reliability across the included studies. Moreover, the Egger test and funnel plot suggest the presence of potential publication bias in this study. This bias could stem from the incorporation of three open-label studies among the included RCTs. This conjecture finds support in the Galbraith plot and Baujat plot.

Additionally, this study conducted a comprehensive analysis of CDK 4/6 inhibitor-related nephrotoxic ADE reports from the FAERS database via OpenVigil. A total of 506 reports implicating CDK 4/6 inhibitors as the primary suspected drugs were identified, with abemaciclib, palbociclib, and ribociclib accounting for 162, 162, and 182 nephrotoxic-related ADE reports, respectively. Notably, the majority of these reports originated from Europe and North America. The incidence of CDK 4/6 inhibitor-related nephrotoxic AEs was found to be higher in the 50–75 age group, likely reflecting the higher prevalence of breast cancer within this demographic. But some research indicates that older women treated with CDK4/6 inhibitors experience a higher rate of severe toxicity compared to younger patients, suggesting that age may influence susceptibility to CDK4/6 inhibitor toxicity.^45^ Therefore, careful consideration of patient’s age is essential. Among the reported PTs for abemaciclib-associated nephrotoxic AEs, “renal impairment”, “renal failure”, and “blood creatinine increased” ranked highest, while for palbociclib and ribociclib, they were “blood creatinine increased”, “acute kidney injury”, and “renal impairment”. Consequently, “blood creatinine increased” emerged as the most commonly reported PT for nephrotoxic AEs associated with these CDK 4/6 inhibitors. Currently, elevated creatinine levels are widely acknowledged as a frequent outcome in HR+, HER2-breast cancer patients undergoing CDK 4/6 inhibitor treatment and are generally considered reversible upon treatment cessation.^46^ However, given the notable incidence of other PTs, elevated creatinine levels may serve as an early indicator of CDK 4/6 inhibitor-induced kidney injury, warranting clinical attention and necessitating appropriate testing or intervention to prevent further renal damage.

Additionally, one case report described an elderly patient with breast cancer with diabetes who developed severe hypoglycemia after using abemaciclib.^47^ Another case mentioned another elderly patient with breast cancer, also with diabetes, who developed lactic acidosis with acute respiratory failure after taking ribociclib.^48^ These two patients were also taking diabetes medications while on CDK4/6 inhibitors. It is speculated that the use of CDK4/6 inhibitors may lead to blood creatinine increased, which in turn may slow down the metabolism of diabetes treatment drugs^45^ and ultimately lead to serious AEs. Thus, the use of CDK4/6 inhibitors should be exercised with extreme caution in patients with underlying diseases who are taking other therapeutic agents at the same time. Potential nephrotoxic AEs may affect blood concentrations of these other drugs, triggering further AEs, which are particularly dangerous for older patients. Although blood creatinine is often used to assess kidney function in clinical practice, it can be affected by a variety of factors. Therefore, cystatin C, a more sensitive and specific indicator of glomerular filtration rate, needs to be introduced. Cystatin C is good at detecting minor glomerular injury and is a highly accurate and specific indicator of early renal function impairment.^49^ Blood creatinine increased had been observed in patients with breast cancer treated with CDK4/6 inhibitors, and cystatin C may be added for renal function assessment to facilitate subsequent treatment formulation.

The results from the FAERS database analysis align with this meta-analysis, reinforcing the notion that the administration of CDK 4/6 inhibitors heightens the likelihood of nephrotoxic AEs in patients with breast cancer. This convergence of evidence from both clinical trial data and real-world observations lends substantial credibility and persuasiveness to the study’s conclusions. The findings aforementioned underscore the importance of vigilant monitoring of renal function during the clinical administration of CDK 4/6 inhibitors to mitigate potential nephrotoxicity.

Among the 17 RCTs we reviewed, 14 studies focused on advanced breast cancer (including 1 study specifically on metastatic breast cancer), while 3 studies targeted early breast cancer. It is indisputable that CDK 4/6 inhibitors are the primary treatment for patients with HR+, HER2-advanced breast cancer. Currently, both abemaciclib and ribociclib have demonstrated efficacy in clinical trials for the treatment of early breast cancer, and abemaciclib has received FDA approval for its adjunctive use in combination with ET, further solidifying its role as a treatment option in early breast cancer.^50^^,^^51^^,^^52^ Two studies offer evidence supporting the adjuvant use of palbociclib in early breast cancer treatment, indicating its substantial anti-proliferative efficacy.^53^^,^^54^ However, findings from PENELOPE-B and PALLAS trials indicate that palbociclib administration did not yield improvements in invasive disease-free survival among patients with early breast cancer.^24^^,^^55^

In conclusion, our findings indicate that both in clinical trials and the real world, CDK 4/6 inhibitors may increase the risk of nephrotoxic AEs in breast cancer treatment, which clinical vigilance is warranted. It is expected that this study will provide guidance for clinical practice.

### Limitations of the study

There are several limitations in this study that warrant consideration. Firstly, among the 17 RCTs included, several were multicenter studies, which might have introduced variations in diagnostic criteria and methodologies for detecting nephrotoxic AEs. Secondly, the studies included were not specifically designed to assess nephrotoxic AEs associated with CDK4/6 inhibitors, potentially resulting in an underestimation of the nephrotoxic AE risk. Furthermore, given that dalpiciclib is the inaugural domestically developed CDK4/6 inhibitor in China and predominantly utilized within China, the absence of pertinent data in the FAERS database impedes the evaluation of nephrotoxic AE incidence in real-world dalpiciclib application scenarios. Despite these limitations, the amalgamation of meta-analysis and FAERS data mining in our study has bolstered the credibility of our investigation into CDK4/6 inhibitor-related nephrotoxic AEs.

## Resource availability

### Lead contact

Further information and requests should be directed to the lead contact Guoshuang Shen (Guoshuangshen@126.com).

### Materials availability

This study did not generate new unique reagents.

### Data and code availability

• All data reported in this paper will be shared by the lead contact upon request.
• All original code has been deposited at Mendeley Data and is publicly available as of the date of publication. DOIs are listed in the key resources table.
• Any additional information required to reanalyze the data reported in this paper is available from the lead contact upon request.

## Acknowledgments

Special thanks are extended to Jinming Li, Hengheng Zhang, and Tianlei Qiu for their invaluable assistance in this study. Funding: None.

## Author contributions

J.C. and J.Z. conceptualized and designed the study; J.C. and J.S. conducted the literature review and performed the statistical analysis; X.Z. and Y.L. collected the data; J.C. drafted the manuscript; G.S. and J.Z. reviewed and edited the manuscript; G.S. supervised the project.

## Declaration of interests

The authors have declared no conflicts of interest.

## STAR★Methods

### Key resources table

**Table:** 

REAGENT or RESOURCE	SOURCE	IDENTIFIER
**Deposited data**		

Raw data and original code	This paper	Mendeley Data：https://doi.org/10.17632/m4s288hg57.1

**Software and algorithms**		

R	Version 4.3.1	http://cran.r-project.org/src/base/R-4
Zotero	Version 6.0.37	http://www.zotero.org

### Method details

#### Data sources

Following the Preferred Reporting Items for Systematic Reviews and Meta-Analyses (PRISMA) statement for trial selection and systematic evaluation,^56^ a comprehensive review of PubMed, Web of Science, and Cochrane Library was conducted up to April 2024. The search terms included: “cyclin-dependent kinase 4/6 inhibitors”, “CDK 4/6 inhibitors”, “abemaciclib”, “palbociclib”, “ribociclib”, “dalpiciclib”, and “breast cancer”.

Additionally, data from the FDA Adverse Event Reporting System (FAERS) database were extracted, by OpenVigil 2.1 (https://openvigil.sourceforge.net) to extract data from the FAERS database and analyzed.^57^ FAERS is a self-reporting system that includes all AEs and medication error information collected by the FDA, providing a large source of real-world data for early identification of AEs.

#### Study selection

Two independent reviewers (Cui and Sun) screened articles containing the keywords in the title and abstract to ensure relevance. Subsequently, the full texts of relevant articles were assessed for eligibility. Inclusion criteria were: (Ⅰ) Randomized controlled phase II and III trials in patients with HR+/HER2-breast cancer, (Ⅱ) subjects assigned to one of four drugs (abemaciclib, palbociclib, ribociclib, and dalpiciclib) or control treatment, and (Ⅲ) event counts or event rates and evaluable sample sizes available for nephrotoxicity-related analysis. Exclusion criteria included case reports, reviews, conference abstracts, cohort studies, phase I studies, single-arm phase II studies, randomized controlled trials (RCTs) with unavailable outcomes, and randomized controlled trials where all groups used CDK 4/6 inhibitors.

#### Data extraction

The trials that allocate patients randomly into CDK 4/6 inhibitor group, placebo group, or control group were included, and relevant information was extracted from each randomized controlled study included. If the relevant results were not reported in the article, data extraction was supported by supplementary materials or ClinicalTrials.gov. In cases of duplicate publication, only the most recent and comprehensive reports containing complete safety data of the clinical trials were included.

The search for “abemaciclib”, “palbociclib”, “ribociclib” and “dalpiciclib” was conducted separately on the OpenVigil 2.1-MedDRA-v24 online analysis website, with the study period limited to inception up to the third quarter of 2023 and a minimum age of 18 years. As of April 2024, there were no AE records related to "dalpiciclib" use in the FAERS database.

### Quantification and statistical analysis

Statistical analysis was conducted using R 4.3.1. The RR and 95% CI were the primary indicators for nephrotoxicity-related AEs. The event counts of nephrotoxicity-related AEs per subject randomized to the CDK 4/6 inhibitor group were compared with those per subject randomized to the control treatment group in each trial. The Higgins inconsistency index (*I*^2^) test with *p* value were used to evaluate heterogeneity. Significant heterogeneity between studies was defined as *p* < 0.05. Specifically, *I*^2^ = 0% indicated no heterogeneity, *I*^2^ ≥ 25% signified mild heterogeneity, *I*^2^ ≥ 50% indicated moderate heterogeneity, and *I*^2^ ≥ 75% represented high heterogeneity. Random effects model was employed for data merging when the *I*^2^ value indicated moderate or high heterogeneity. Conversely, common effect model was applied when heterogeneity was low.

The data obtained from the FAERS database is organized, consolidated, and deduplicated. Abemaciclib or palbociclib or ribociclib were classified as “primary suspects” and the data was categorized by patient gender, age and country. In this study, proportional reporting ratio (PRR) and reporting odds ratio (ROR) methods were used to analyze the cleaned data, which are widely used in AE signal detection.
