# Supplementary material for: Association between cyclin-dependent kinase 4/6 inhibitors and nephrotoxicity in patients with breast cancer: A Systematic Review and meta-analysis
Source: iScience. 2024 Nov 12;27(12):111370. doi: 10.1016/j.isci.2024.111370 (PMC11618030; doi:10.1016/j.isci.2024.111370)
Supplement: Document S1. Figures S1–S9 [file mmc1.pdf]

iScience, Volume *27*

Supplemental information

Association between cyclin-dependent kinase 4/6 inhibitors and nephrotoxicity in patients with breast cancer: A Systematic Review and meta-analysis

Jiayong Cui, Jinquan Sun, Xueying Zhou, Yi Li, Jiuda Zhao, and Guoshuang Shen

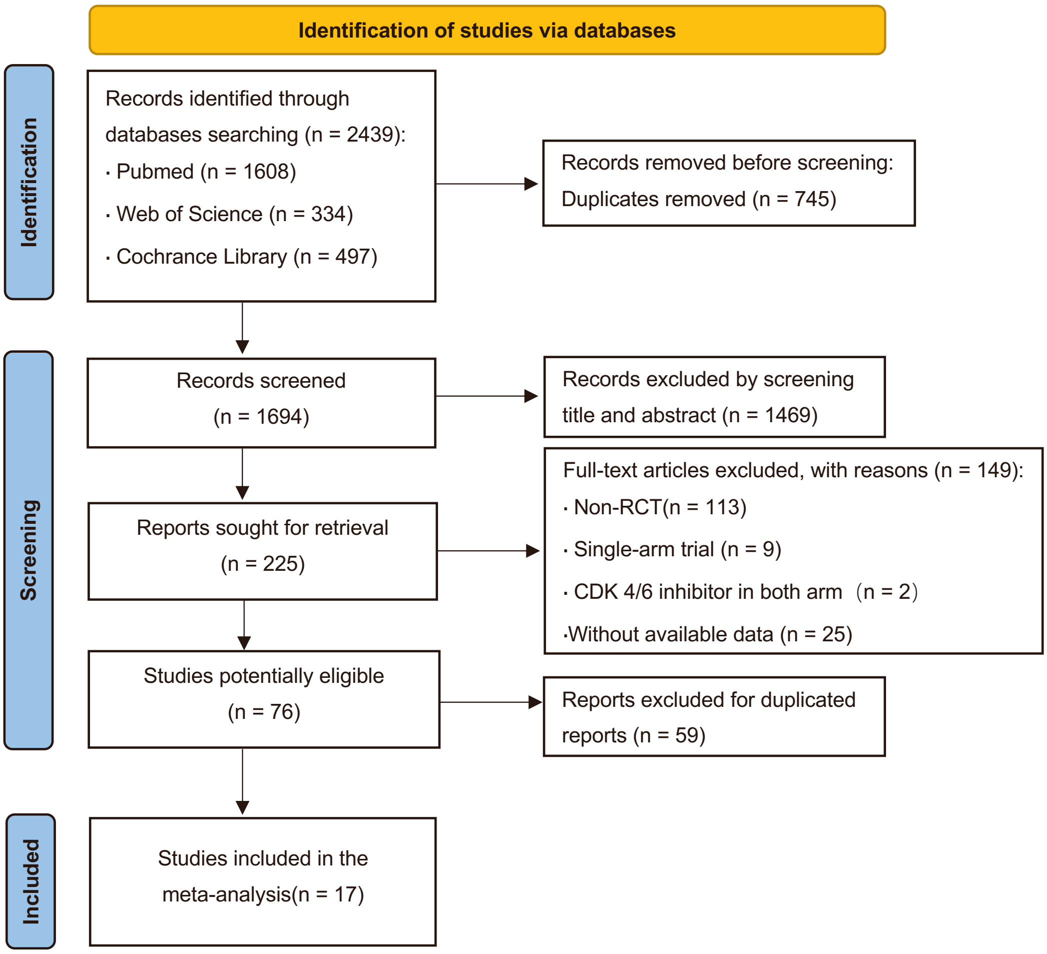

Figure S1. Flow diagram of eligible studies. Non-RCT: Non-Randomized Controlled Trial; CDK: cyclin-dependent kinases.

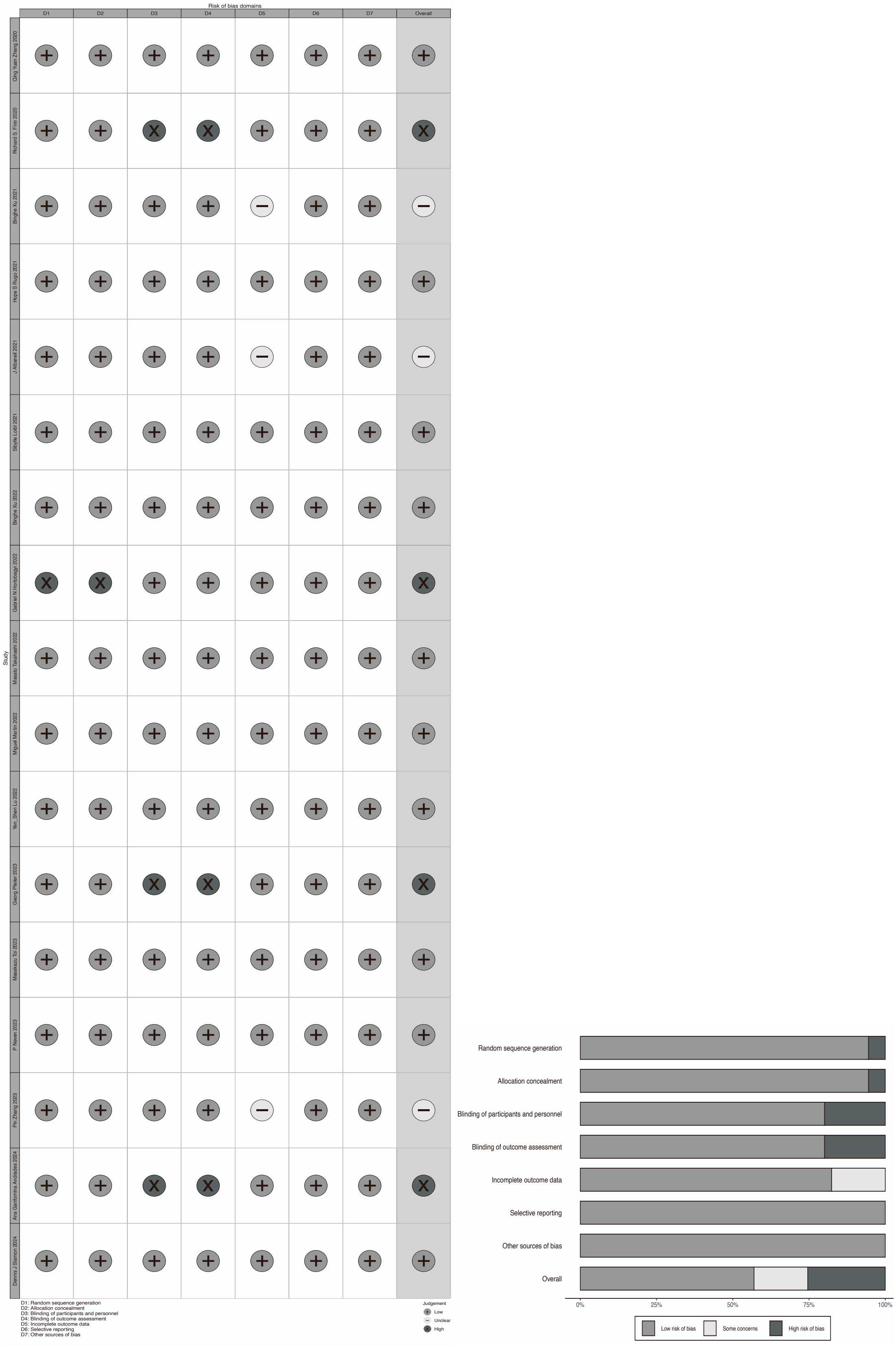

Figure S2. Risk of bias of included studies.

Study	RR	95%−CI	P−value	Tau2	Tau	I^2
Omitting Qing Yuan Zhang 2020	3.01	[2.01; 4.53]	< 0.01	0.5085	0.7131	87%
Omitting Richard S. Finn 2020	3.29	[2.20; 4.92]	< 0.01	0.4959	0.7042	88%
Omitting Binghe Xu 2021	3.13	[2.07; 4.75]	< 0.01	0.5334	0.7303	88%
Omitting Hope S Rugo 2021	3.11	[2.06; 4.70]	< 0.01	0.5283	0.7268	88%
Omitting J Albanell 2021	3.20	[2.11; 4.87]	< 0.01	0.5376	0.7332	88%
Omitting Sibylle Loibl 2021	3.38	[2.27; 5.01]	< 0.01	0.4535	0.6734	85%
Omitting Binghe Xu 2022	3.07	[2.04; 4.61]	< 0.01	0.5148	0.7175	88%
Omitting Gabriel N Hortobagyi 2022	3.04	[2.01; 4.58]	< 0.01	0.5177	0.7195	87%
Omitting Masato Takahashi 2022	3.01	[2.00; 4.55]	< 0.01	0.5134	0.7165	87%
Omitting Miguel Martin 2022	3.35	[2.24; 5.01]	< 0.01	0.4743	0.6887	86%
Omitting Yen−Shen Lu 2022	3.17	[2.09; 4.82]	< 0.01	0.5370	0.7328	88%
Omitting Georg Pfeiler 2023	3.28	[2.17; 4.98]	< 0.01	0.5191	0.7205	87%
Omitting Masakazu Toi 2023	2.91	[1.99; 4.26]	< 0.01	0.4447	0.6668	87%
Omitting P Neven 2023	3.18	[2.08; 4.84]	< 0.01	0.5425	0.7366	88%
Omitting Pin Zhang 2023	3.12	[2.06; 4.75]	< 0.01	0.5375	0.7332	88%
Omitting Ana Ganfornina Andrades 2024	2.58	[1.88; 3.52]	< 0.01	0.2318	0.4815	69%
Omitting Dennis J Slamon 2024	3.22	[2.12; 4.89]	< 0.01	0.5335	0.7304	88%
**Random effects model**	**3.12**	**[2.11; 4.63]**	**< 0.01**	**0.4977**	**0.7055**	**87%**

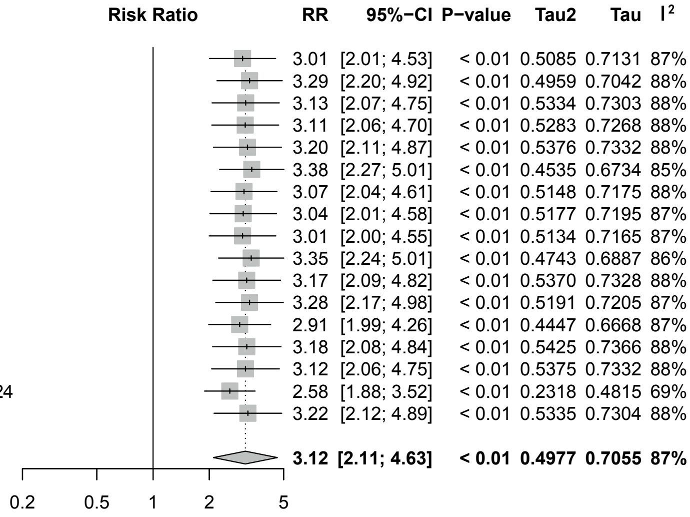

Figure S3. Sensitivity analysis of included studies. RR: Risk Ratio.

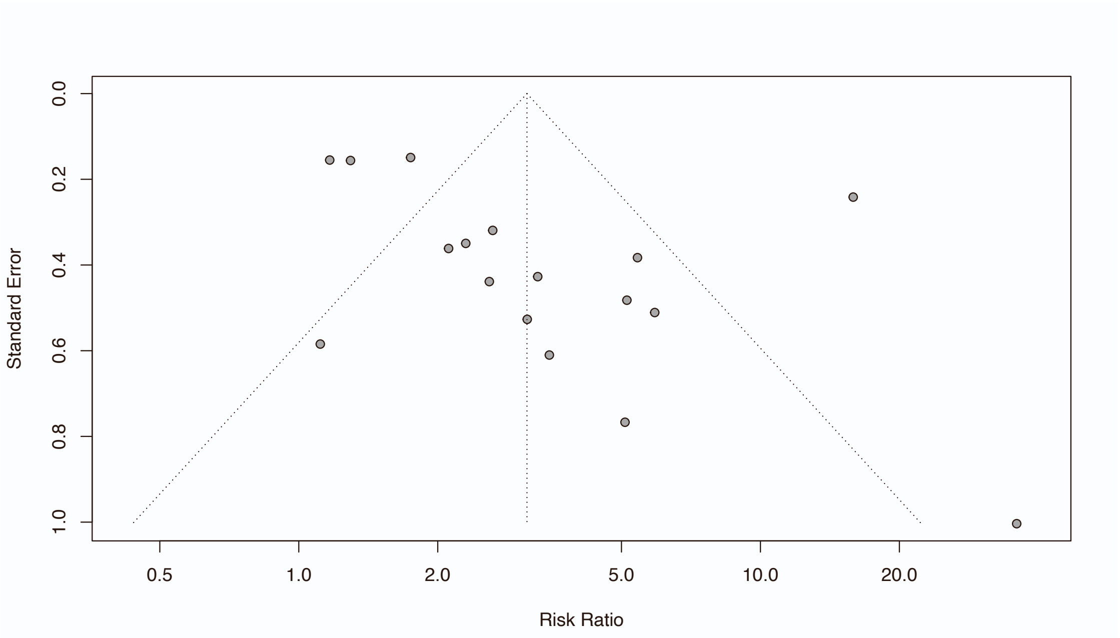

Figure S4. Funnel plot of included studies.

```
> metabias(m1,method.bias="Egger",plotit=TRUE)
Linear regression test of funnel plot asymmetry

Test result: t = 2.24, df = 15, p-value = 0.0406
Bias estimate: 2.6583 (SE = 1.1861)

Details:
- multiplicative residual heterogeneity variance (tau^2 = 6.1255)
- predictor: standard error
- weight:    inverse variance
- reference: Egger et al. (1997), BMJ
> 
```

Figure S5. The Egger method detects the output of publication bias.

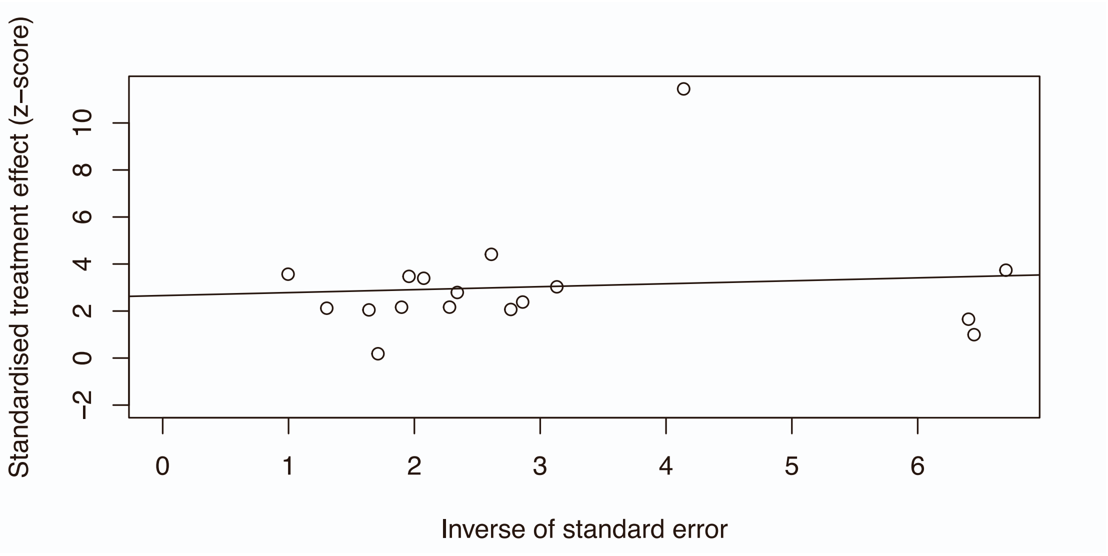

Figure S6. Egger funnel plot.

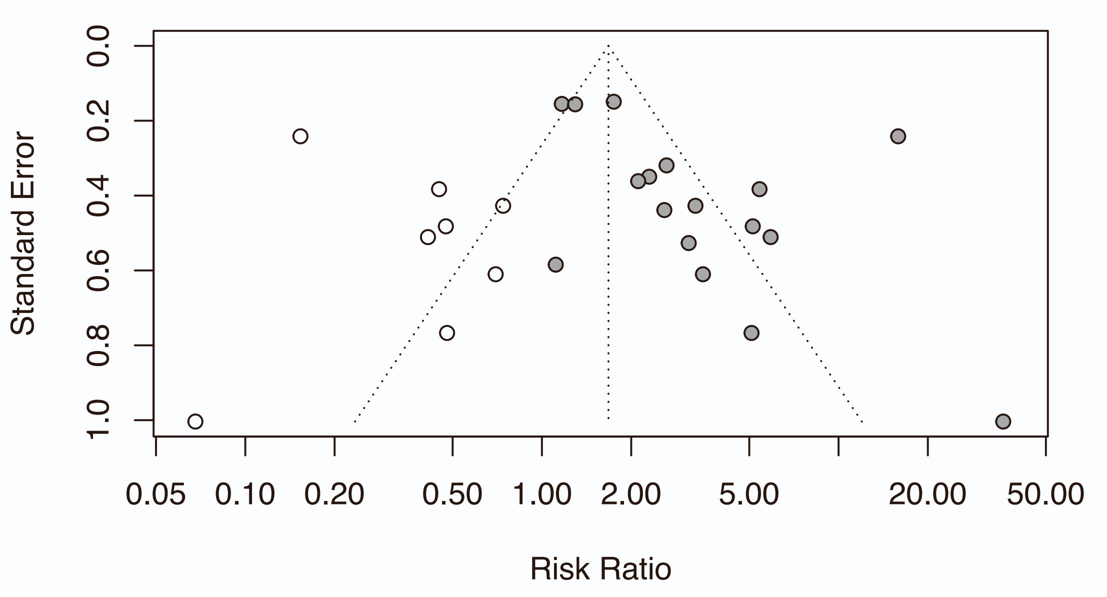

Figure S7. Funnel plot after imputation.

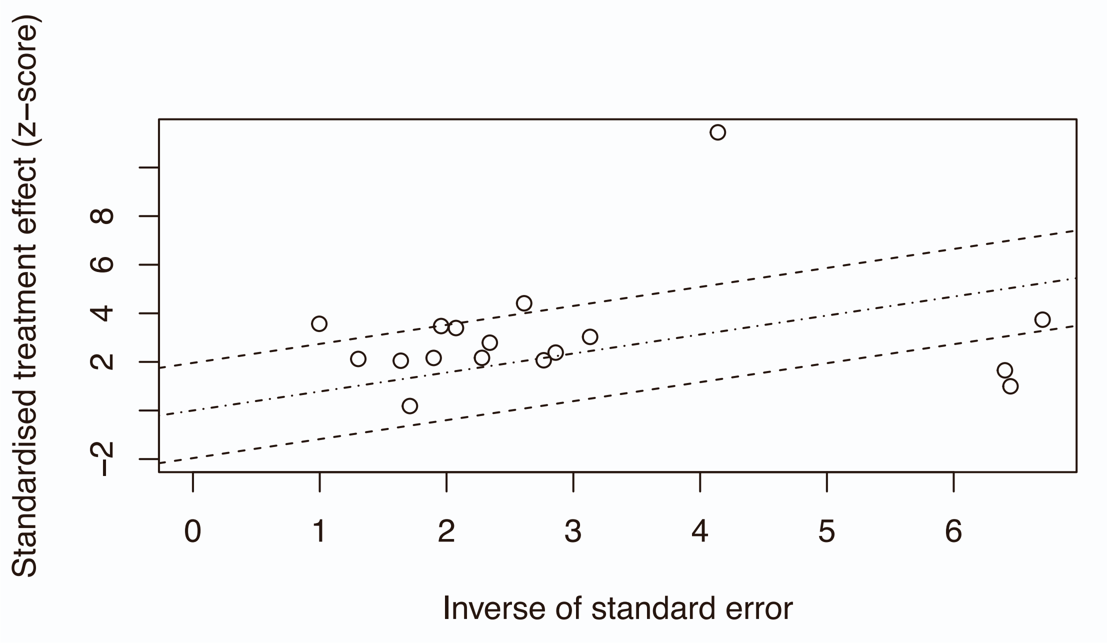

Figure S8. Galbraith plot of included studies.

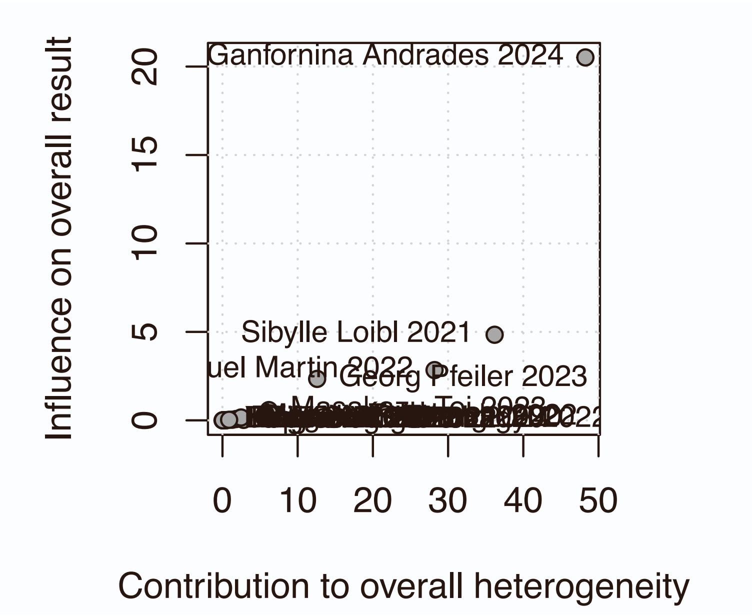

Figure S9. Baujat plot of included studies.
